# Sex differences in self-regulation: an evolutionary perspective

**DOI:** 10.3389/fnins.2014.00233

**Published:** 2014-08-04

**Authors:** Niki Hosseini-Kamkar, J. Bruce Morton

**Affiliations:** Cognitive Development and Neuroimaging Laboratory, Department of Psychology, The University of Western OntarioLondon, ON, Canada

**Keywords:** sex differences in self-regulation, parental investment theory, sex differences in inhibition, sex differences in impulsivity, sex differences the mesocorticolimbic dopamine pathway, delay-discounting, delay of gratification, inhibitory control

## Abstract

Bjorklund and Kipp (1996) provide an evolutionary framework predicting that there is a female advantage in inhibition and self-regulation due to differing selection pressures placed on males and females. The majority of the present review will summarize sex differences in self-regulation at the behavioral level. The neural and hormonal underpinnings of this potential sexual dimorphism will also be investigated and the results of the experiments summarized will be related to the hypothesis advanced by Bjorklund and Kipp (1996). Paradoxically, sex differences in self-regulation are more consistently reported in children prior to the onset of puberty. In adult cohorts, the results of studies examining sex differences in self-regulation are mixed. A few recent experiments suggesting that females are less impulsive than males only during fertile stages of the menstrual cycle will be reviewed. A brief discussion of an evolutionary framework proposing that it is adaptive for females to employ a self-regulatory behavioral strategy when fertile will follow.

## An evolutionary perspective on sex differences in self-regulation

Self-regulation is the capacity to select actions that lead to favorable outcomes and avoid actions that lead to unfavorable outcomes. Bjorklund and Kipp (1996) examine the hypothesis that males and females differ in self-regulation and in their ability to inhibit responses due to the different selection pressures placed on male and female hominids. According to parental investment theory, males and females invest differentially in their offspring. Specifically, human females have only a few high quality gametes (relative to males), they are required to provide offspring with nutrients for an extended period of time during pregnancy; furthermore, they are also required to provide nutrients and care for offspring for many years after pregnancy. All of these require a large amount of resources in terms of Darwinian fitness (Trivers, 1972). On the other hand, males have many gametes, they are not required to invest resources in terms of time or nutrients to their offspring directly and as a result, they invest much less in each individual offspring. One consequence of parental investment theory is that females are required to be more selective in terms of securing a mate that is capable of providing resources (for both herself and her offspring) for an extended period of time. In contrast, males are not required to be as discriminatory in terms of selecting mates—after all, they have many gametes to disseminate (Trivers, 1972). In other words, males and females have evolved differential strategies for effective mate selection—with females being more selective.

However, Bjorklund and Kipp (1996) propose that the reproductive success of females depends both on the ability to select high quality mates and also on the ability to inhibit maladaptive social and sexual responses. In the context of sexual selection, an example of a maladaptive response may simply be “selecting” lower quality mates. Bjorklund and Kipp (1996) claim that in order to satisfy the needs of helpless offspring, females must frequently delay gratifying their own needs in order to meet the needs of their offspring. Furthermore, females must inhibit aggressive responses to aversive stimuli including misbehaving children who often transgress the boundaries set by their parents (Bjorklund and Kipp, 1996). According to Bjorklund and Kipp's (1996) hypothesis, due to the different selection pressures placed on males and females, females should have evolved a greater ability to inhibit prepotent responses. To test this hypothesis, the authors reviewed literature on sex differences in inhibitory control from a variety of domains including behavioral inhibition, social inhibition, and cognitive inhibition. The authors concluded that overall, there is a moderate female advantage in behavioral inhibition, and a strong female advantage in social inhibition (Bjorklund and Kipp, 1996). Therefore, the results confirm the hypothesis that females have a greater capacity to delay gratification and inhibit prepotent responses relative to males—especially in the domain of social inhibition. While Bjorklund and Kipp (1996) conclude that the result of their review presents substantial evidence that there is a female advantage in inhibition and self-regulation, others have obtained conflicting results (See Table 1). The present paper will summarize research on sexual dimorphisms in self-regulation at the behavioral level. Also, the degree to which these sex differences are due to sexual dimorphisms within the mesocorticolimbic dopamine pathway (MCLP) will be investigated. Furthermore, the role of sex hormones and how they may alter the functioning of the MCLP and subsequent behavior will be briefly reviewed. The conclusions drawn from the research reviewed will be summarized within the context of Bjorklund and Kipp's (1996) evolutionary framework.

**Table 1:** **Sexual dimorphisms in inhibition at the behavioral level**.

**References**	**Study type**	**Sample characteristics**	**Measure of inhibition/self-control**	**Results/conclusions**
Mischel and Underwood, 1974	Empirical	*n* = 80 preschool children (48 F and 32 M)	Delay of gratification paradigm	Females waited significantly longer than males
Kochanska et al., 2000	Longitudinal study	*n* = 106; Caucasian	Large battery of tasks to measure effortful control	A sex difference (female advantage) in many of the tasks used to measure effortful control
Silverman, 2003	Meta-analysis	38 effect sizes obtained from 33 studies	Studies that investigated delay of gratification measures	A small female advantage in delay of gratification
Duckworth and Seligman, 2006	Empirical	*n* = 140.8th grade students	A composite self-discipline score from a variety of questionnaires	Girls in the 8th grade were more self-disciplined than boys
Else-Quest et al., 2006	Meta-analysis	189 studies were included	Empirical studies that tested for temperamental differences between boys and girls	A moderate sex difference in inhibitory control favoring girls
Reynolds et al., 2006	Empirical	*n* = 70 healthy adult participants	Personality inventory measures and behavioral tasks including the DD task	On the delay-discounting measure women performed more impulsively (discounted more steeply)
Yuan et al., 2008	Empirical	*n* = 30 healthy adults (15 F, 15 M)	Behavioral inhibitory control measured using a modified Oddball Task and ERPs were recorded	Women were faster at responding to deviant stimuli (perhaps a greater sensitivity to deviant stimuli which may enhance performance in inhibitory control)
Matthews et al., 2009	Empirical (5 years longitudinal study)	*n* = 268 kindergarteners when they began the study	Self-regulation was measured both directly (behaviorally) and indirectly (rating scales administered to teachers)	Girls outperformed boys on both direct and indirect measures of self-regulation
Beck and Triplett, 2009	Empirical	*n* = 387 adult participants in Test 1; *n* = 299 of the above individuals participated again in Test 2	The delay-discounting task	Women discounted more steeply than men
Cross et al., 2011	Meta-analysis	277 studies were included and 741 *d* values were calculated	Aim was to investigate sex differences in impulsivity	Women were more sensitive to punishments and men scored higher on risk taking and sensation seeking. Did not confirm a sex difference in DD with females discounting more steeply
Liu et al., 2012	Empirical	*n* = 28 (15 F, 13 M) healthy adults	Personality trait measures, Impulse inhibition task, and fMRI data were obtained	Females demonstrated lower impulsivity scores on only one measure of impulsivity: Zuckerman-Kuhlman Personality Questionnaire (ZKPQ)
Weafer and de Wit, 2013	Review	–	–	Lab animals and humans: females discount more steeply.
				Lab animals: males demonstrate more impulsive action
				In humans: sex differences in impulsive action depend on the sample and tasks used
Weis et al., 2013	Empirical	*n* = 53 (34 F, 19 M) German 5th graders	Self-regulation and emotional-regulation were measured	A female advantage in behavioral self-regulation
Thakkar et al., 2014	Empirical	*n* = 631 (346 F, 285 M) healthy adults	Stop signal task	No sex differences in overall accuracy/response inhibition, but women showed greater sensitivity to trial history. Suggests a more flexible adjustment strategy to speed-accuracy trade-offs in women

## Behavioral sexual dimorphisms

### Delay of gratification

In the 1970s, Walter Mischel created the delay of gratification paradigm to test children's ability to resist temptation and forgo a small immediate reward in order to obtain a larger delayed reward. The paradigm consists of preschool children being asked to observe one marshmallow, without touching or eating it. The research assistant leaves the child alone and if the child can resist the temptation to touch the marshmallow and is able to wait until the research assistant returns voluntarily, the child is given the opportunity to have two marshmallows. This elegant and simple paradigm allows researchers to test children's ability to select a large delayed reward in favor of small immediate reward (Mischel and Underwood, 1974). Presumably, children who are capable of waiting and resisting the temptation of the small immediate reward have higher self-control and are better able to delay gratification.

According to the hypothesis proposed by Bjorklund and Kipp (1996), one might expect that females will be better able to delay gratification and resist a small immediate reward in comparison to males. Interestingly, Mischel and Underwood (1974) were the first to report a sex difference in delay of gratification. The authors reported that female preschoolers were able to wait for significantly longer periods of time to obtain the larger reward in comparison to their male counterparts—these results confirm the predictions proposed by Bjorklund and Kipp (Mischel and Underwood, 1974). More recently, a meta-analysis was conducted on experiments using the delay of gratification paradigm with the intention of directly testing Bjorklund and Kipp's (1996) hypothesis that there is a female advantage in inhibitory control. Silverman (2003) investigated 33 studies that required participants to make a choice between a small immediate reward and larger delayed reward. The results of the meta-analysis revealed a statistically significant female advantage in the capacity to delay gratification. Silverman (2003) concludes that the effect size of the female advantage in delay of gratification is relatively small; however, this may be due to instruments that lack precision and small sample sizes. Importantly, the author states that these results provide substantial support for the hypothesis proposed by Bjorklund and Kipp (Silverman, 2003). Based on these findings, it appears as though females are better suited to delay gratification and resist temptation in comparison to males and that this sex difference emerges early in development.

#### Self-control

Duckworth and Seligman (2006) conducted an experiment to test whether or not there are sex differences in self-control in 8th grade children. Self-control was defined as the ability to inhibit prepotent responses in service of achieving a higher goal—furthermore, the authors specified that self-control would not occur automatically and requires conscious effort (Duckworth and Seligman, 2006). To measure self-control the authors used a composite self-discipline score from self-report, parent and teacher questionnaires, in addition to a delay of gratification measure. According to the authors, a comprehensive battery of assessments is a more reliable measure of self-control than any one component measure alone. Consistent with Bjorklund and Kipp's (1996) hypothesis, the composite score for self-control demonstrated a significant sex difference favoring girls. It is important to note however, that the smallest effect size for the sex difference was observed in the delay of gratification measure (Duckworth and Seligman, 2006). Matthews et al. (2009) also investigated sex differences in self-regulation in kindergarten children. In this case, self-regulation was defined as behavioral regulation which encompasses working memory, attentional control, switching, and inhibitory control (Matthews et al., 2009). Self-regulation was measured both directly with the use of the Head-Toes-Knees-Shoulders task and indirectly through the use of the Child Behavior Rating Scale (CBSR). The results confirmed that girls outperformed boys on both measures of self-regulation (Matthews et al., 2009). More recently, Weis et al. (2013) also reported a significant sex difference favoring girls in behavioral self-regulation in a sample of German 5th graders. In this case, behavioral self-regulation was measured using the Self-Control Scale (SCS-K-D). Overall, these results confirm that early in development, girls appear to outperform boys on measures of self-regulation.

#### Delay-discounting

While the results of delay of gratification measures and teacher/parent reports of self-regulation seem to indicate that there is a female advantage in self-control (at least early in development), the results on a related measure (delay discounting) are contradictory. Delay discounting is a task used to measure impulsive choice by asking participants to select between a large reward delivered at variable delays (1 day, 7 days, 30 days etc.) and a smaller immediate reward. As the length of the delayed reward increases, participants are more inclined to select the smaller immediate reward. However, there is variability in how quickly participants discount the value of the larger delayed reward and this variation can be used as a measure of impulsive choice; in other words, greater discounting is indicative of impulsive choice (Reynolds and Schiffbauer, 2004). Some evolutionary theorists have proposed that sex differences in impulsivity are the result of selection pressures in the context of male intra-sexual competition. For example, MacDonald (2008) states that males are more likely to score higher on behavioral approach measures (sensation seeking, impulsivity, reward seeking, and aggression) and are thus less likely to control prepotent approach tendencies. According to the hypotheses of Bjorklund and Kipp (1996) and MacDonald (2008), it would be expected that males would discount more steeply compared to females. Dittrich and Leipold (2014) provide evidence that supports this hypothesis; they report a sex difference in time preference with males preferring a smaller immediate payment rather than a larger delayed payment. Interestingly, the authors explicitly state that their findings provide direct support for Bjorklund and Kipp's (1996) evolutionary hypothesis suggesting that females are better able to delay gratification in comparison to males (Dittrich and Leipold, 2014).

However, many other researchers have observed the opposite pattern of sex differences in relation to delay-discounting—with females discounting more steeply than males (Reynolds et al., 2006; Beck and Triplett, 2009). A review article investigating sex differences in impulsive choice (which refers to the tendency to prefer small immediate rewards rather than large delayed rewards) and impulsive action (a lack of behavioral inhibition) demonstrates that many of these inconsistent findings may be due to variations in the subjects under study and the tasks used (Weafer and de Wit, 2013). For example, Weafer and de Wit's review article demonstrates that in humans and some other species, females tend to discount more steeply than males on measures of delay-discounting (impulsive choice). However, in rodents, males exhibit a greater tendency toward impulsive action (a lack of behavioral inhibition). In humans, sex differences in impulsive action depend on the task administered. For example, when participants are administered the Go/No-Go task, males tend to commit more inhibitory errors (failing to inhibit a prepotent response) than females. In contrast, when participants are administered the Stop Signal Task, women tend to commit more inhibitory errors than males (Weafer and de Wit, 2013). Based on these findings and others (see Table 1) it seems as though females have an advantage in terms of delay of gratification; however, the results using different measures of impulsivity and behavioral inhibition are less consistent. In addition, one pattern of findings that emerges from the studies summarized above is that the sex difference in delay of gratification and inhibitory control more generally, seems to exist in childhood. However, the studies that investigated sex differences in inhibitory control and delay discounting in adulthood are less consistent—with some studies indicating that females discount more steeply and others demonstrating that females score lower on questionnaire measures of impulsivity (See Table 2). One potential reason for these mixed findings in adult cohorts could be due to the activational effects of hormones (other reasons may include cultural factors, developmental factors, and methodological factors). Sex hormones may modulate the neural circuitry underlying self-regulation and result in differential patterns of behavior across different phases of the menstrual cycle. In other words, the mixed findings in self-regulation in adult cohorts could potentially be due to variations in levels of hormones exerting their influence on the MCLP.

**Table 2:** **Sexual dimorphisms at the behavioral level: children vs. adults**.

**References**	**Children/adults**	**Measure**	**Conclusions**
Mischel and Underwood, 1974	Children (*n* = 80)	Delay of gratification	Females waited significantly longer than males
Kochanska et al., 2000	Children (*n* = 106)	Large battery of tasks to measure effortful control	These results provide evidence for a female advantage in effortful control
Duckworth and Seligman, 2006	Children (*n* = 140)	Composite self-discipline score	Girls in the 8th grade were more self-disciplined than boys
Else-Quest et al., 2006	Children—meta-analysis of studies that investigated sex differences in temperament in children	Meta-analysis of empirical studies	The moderate sex difference in inhibitory control suggests that girls are better able to control inappropriate responses and behaviors in comparison to boys
Matthews et al., 2009	Children (*n* = 268)	Self-regulation (measured directly and indirectly)	Girls outperformed boys on both direct and indirect measures of self-regulation
Weis et al., 2013	Children (*n* = 53)	Self-regulation (the self control scale)	In a sample of German 5th graders, there appears to be a female advantage in behavioral self-regulation
Silverman, 2003	Unclear (perhaps both)	Meta-analysis of studies assessing delay of gratification measures	Overall, there appears to be a small female advantage in delay of gratification
Reynolds et al., 2006	Adults (*n* = 70)	Numerous measures including delay-discounting	On the delay-discounting measure women performed more impulsively than men (discounted more steeply)
Yuan et al., 2008	Adults (*n* = 30)	Behavioral inhibitory control (measured using the Oddball Task)	Women are faster at responding to deviant stimuli in an Oddball Task compared to men
Beck and Triplett, 2009	Adults (*n* = 387/*n* = 299)	Delay-discounting task	Women discounted more steeply than men
Cross et al., 2011	Unclear (perhaps both)	Meta-analysis of studies investigating sex differences in impulsivity	Women were more sensitive to punishments and men scored higher on risk taking and sensation seeking
Liu et al., 2012	Adults (*n* = 28)	Personality measures of impulsivity and Go/No-Go task	Females demonstrated lower impulsivity scores on only one measure of impulsivity (ZKPQ)
Thakkar et al., 2014	Adults (*n* = 631)	Measured response inhibition and response monitoring using the Stop Signal Task	No sex differences in overall accuracy or response inhibition, but women showed greater sensitivity to trial history

## Biological mechanisms

### Neural sexual dimorphisms in the mesocorticolimbic dopamine pathway

Sex differences in the structure and function of the MCLP might provide some insight into the inconsistent findings on self-regulation and inhibition at the behavioral level. Much of the research investigating sex differences in the MCLP focuses on reward-related activity primarily because the MCLP is involved in rewarding and motivated behaviors. It is conceivable that individuals who have increased neural responses to rewards and/or are more sensitive to the prospect of obtaining rewards would have greater difficulty inhibiting prepotent responses and delaying gratification.

The MCLP originates in the ventral tegmental area (VTA) and has projections to the nucleus accumbens (NAc), bed nucleus of stria terminalis (BNST), and the frontal cortex. GABAergic, glutamatergic, and opioid peptidergic systems modulate activity in the VTA and NAc (Bobzean et al., 2014). Furthermore, dopaminergic transmission within the MCLP modulates rewarding and motivated behaviors. Sexual dimorphisms within the structure and function of the MCLP have been well documented in both human and animal samples (See Table 3). For example, a recent Magnetic Resonance Imaging (MRI) study that investigated sexual dimorphisms in the basal ganglia revealed that sexual dimorphisms exist in some structures of the basal ganglia (globus pallidus and putamen) but not others (caudate nucleus and nucleus accumbens) (Rijpkema et al., 2012). Andersen et al. (1997) also investigated structural sex differences in the MCLP using audioradiography and demonstrated developmental sexual dimorphisms in D1 and D2 receptors the striatum of male and female rats. Activation of D1 receptors ultimately increased cyclic adenosine monophosphate (cAMP), whereas activation of D2 receptors indirectly reduced cAMP concentrations. Specifically, males had enhanced overproduction and elimination of D1 and D2 receptors relative to females, and males sustained the greater D1 overproduction in the nucleus accumbens into adulthood (Andersen et al., 1997). Evidently, structural sexual dimorphisms exist in the development of the MCLP.

**Table 3:** **Sexual dimorphisms in the MCLP**.

**References**	**Study type**	**Sample characteristics**	**Measures**	**Results/conclusions**
Andersen et al., 1997	Empirical	Male and female Sprague-Dawley rats (*n* = 48)	D1 and D2 receptors assessed using autoradiography Analysis conducted on post-mortem brains	Males had greater overproduction and elimination of D1 and D2 receptors in the striatum relative to females. Males also had greater D1 receptor overproduction in the nucleus accumbens (sustained into adulthood)
Pohjalainen et al., 1998	Empirical	Healthy adult Finnish volunteers (*n* = 54, 21 F, 33 M)	Investigated striatal D2 receptor density, affinity, and binding potential using PET and [^11^C]raclopride	Women had lower D2 receptor affinity in the striatum. However, postmenopausal women had higher D2 receptor affinity than men
Laakso et al., 2002	Empirical	*n* = 35 (12 F, 23 M) healthy adults	Synaptic concentrations of dopamine in the striatum using PET and [^18^F]fluorodopa	Women have a higher striatal dopamine synthesis capacity than men and there is a decrease in dopamine activity with age in men but not women
Munro et al., 2006	Empirical	*n* = 43 (15 F, 28 M) healthy adults	Magnitude of dopamine, subjective, and neuroendocrine responses to amphetamine. Tested using PET and [^11^C]raclopride in the ventral striatum	Men had greater dopamine release in the ventral striatum. Women in the luteal phase of the menstrual cycle had lower baseline D2 receptor binding potential
Dreher et al., 2007	Empirical	*n* = 13 healthy women	Investigated the role of estrogen and progesterone on the reward system using fMRI during different phases of menstrual cycle	Enhanced activity of the reward system during the mid-follicular phase (when estrogen is not opposed by progesterone)
Robinson et al., 2010	Empirical	*n* = 29 (15 F, 14 M) healthy adults	Examined the influence of artificially reducing dopamine on performance on a reversal learning reward and punishment task	Depletion of dopamine improves punishment-based reversal learning while leaving reward-based reversal learning unaffected—only observed in females
Martin-Soelch et al., 2011	Empirical	*n* = 24 (12 F, 12 M) healthy adults	Striatal dopamine response to unpredictable monetary rewards using PET and [^11^C]raclopride	Dopamine release in response to unpredictable rewards is lateralized in males
Rijpkema et al., 2012	Empirical	Cohort 1 *n* = 463 (261 F, 202 M);Cohort 2 *n* = 541 (330 F, 211 M)	Investigated sexual dimorphisms in the basal ganglia using MRI	Sexual dimorphisms exist in some structures of the basal ganglia (glubus pallidus and putamen) but not others (caudate nucleus and nucleus accumbens)

In addition to the above purely structural studies, numerous other research groups have investigated both structural and functional sex differences. For example, Pohjalainen et al. (1998) investigated D2 receptor density (B_max_), affinity (K_d_), and binding potential (B_max_/K_d_) using positron emission tomography (PET) and [^11^C]raclopride (a D2 receptor radioligand) in healthy adult volunteers. Their results indicate that generally, women have lower D2 receptor affinity in the striatum compared to men. The lower D2 receptor affinity suggests an increase in endogenous striatal dopamine concentrations in women (Pohjalainen et al., 1998). Laakso et al. (2002) used PET and [^18^F]fluorodopa (an analog of L-DOPA which is the immediate precursor of dopamine) and confirmed that women have higher striatal dopamine synthesis capacity than men; furthermore, there was an effect of aging such that there was a decrease in dopamine activity with age in men but not women (Laakso et al., 2002). Taken together, these results suggest that males have higher D1 receptors in the nucleus accumbens (Andersen et al., 1997); women may have lower D2 receptor affinity compared to men (Pohjalainen et al., 1998), and this reduced D2 receptor affinity may result in higher striatal dopamine synthesis capacity in women relative to men (Laakso et al., 2002). These sexual dimorphisms in the structure and subsequent function of the MCLP may make men and women differentially susceptible to the reinforcing properties of rewards. To investigate this hypothesis directly, Munro et al. (2006) investigated sex differences in the magnitude of dopamine release, and subjective and neuroendocrine responses to amphetamine using PET and [^11^C]raclopride. In stark contrast to the findings reported by Laakso et al. (2002), the results of the Munro et al. (2006) study revealed that men had greater dopamine release in the ventral striatum in response to amphetamines. Perhaps under baseline conditions, females have greater dopamine synthesis capacity (Laakso et al., 2002); however, when administered a rewarding substance, males have greater dopamine release in the ventral striatum (possibly a compensatory effect). Furthermore, Munro et al. (2006) report that the baseline binding potential of D2 receptors varies as a function of the menstrual cycle: women in the luteal phase had lower baseline binding potential in the putamen relative to women in the follicular phase (Munro et al., 2006). In the light of these findings, an investigation of hormonal influences on the MCLP is warranted.

### Hormonal influences

To explore the possibility that the MCLP is modulated by gonadal hormones in humans, Dreher et al. (2007) obtained functional Magnetic Resonance Imaging (fMRI) scans from healthy women during the luteal and mid-follicular phases of their menstrual cycle. The fMRI paradigm included an event-related reward task; the results provide evidence of enhanced blood-oxygen-level-dependent (BOLD) responses associated with rewards during the mid-follicular phase of the menstrual cycle (when estrogen is not opposed by progesterone; Dreher et al., 2007). These results suggest that females respond more robustly to rewards during the phase of their menstrual cycle directly preceding ovulation. Furthermore, the results from the Dreher et al. (2007) neuroimaging study are in contrast to findings of hormonal modulation of impulsivity at the behavioral level. For example, Carroll et al. (2013) conducted an experiment that illustrates that rhesus monkeys self-administered higher levels of phencyclidine (PCP) during the luteal (vs. follicular) phase of the menstrual cycle and moreover, impulsive choice (on a delay-discounting measure administered to the same subjects) was greater during the luteal phase of the menstrual cycle. Smith et al. (2014) reported similar findings in a group of healthy adults who were administered a delay-discounting task. The results of their experiment revealed that the preference for an immediate reward declined during the middle of the menstrual cycle when fertility peaks. Similarly, Khaighobadi and Stevens (2013) administered the delay-discounting task to healthy females before and after exposure to either attractive male faces or neutral landscapes. The authors reported an interaction between fertility status and image type, such that women who were fertile and who had viewed images of attractive men chose the small, immediate reward less often than women at peak fertility viewing neutral images. The researchers conclude that women at peak fertility became less impulsive and that this was contradictory to their original hypothesis (Khaighobadi and Stevens, 2013). Interestingly, these results provide indirect evidence for the predictions proposed by Bjorklund and Kipp (1996). Due to the fluctuating levels of estrogen and progesterone across the menstrual cycle, females may adopt differential strategies to facilitate reproduction and mate selection during different phases of their menstrual cycle. For example, being impulsive might not incur enormous risks during phases of the menstrual cycle when females are not fertile—any risk incurred would only apply to the female herself and not to a developing embryo. In accordance with this hypothesis, Pine and Fletcher (2011) demonstrated that women in their luteal phase (non-fertile phase) are more impulsive than women in other phases of the menstrual cycle. However, according to parental investment theory, females must be selective in securing an appropriate mate and therefore, they may exhibit less impulsivity during periods of their menstrual cycle when they have the capacity to reproduce (Carroll et al., 2013; Khaighobadi and Stevens, 2013; Smith et al., 2014). These fluctuations as a result of hormonal changes during the menstrual cycle may help explain the mixed findings in delay-discounting in adult cohorts. For instance, the studies that indicate that women discount more steeply may have investigated women in non-fertile phases of the menstrual cycle. Conversely, the research that indicates that women are less impulsive may have inadvertently recruited females in the fertile phase of their menstrual cycle.

## Concluding remarks

There are a few recent studies that suggest that females are less impulsive during fertile phases of the menstrual cycle (Pine and Fletcher, 2011; Carroll et al., 2013; Khaighobadi and Stevens, 2013; Smith et al., 2014) and these findings provides indirect support for the Bjorklund and Kipp (1996) predictions. It may not be the case that females are constantly more self-controlled and less impulsive than males. In other words, sex differences in self-control are not trait-like enduring dimorphisms but rather, these sex differences fluctuate with the changing hormonal environment of the female menstrual cycle. It appears as though females who are high in estrogen (during the fertile phase of the menstrual cycle) are generally less impulsive than men. If this is the case, then support is provided for the hypothesis that at least during potentially reproductive periods, females employ a more self-controlled and less impulsive behavioral strategy.

### Conflict of interest statement

The authors declare that the research was conducted in the absence of any commercial or financial relationships that could be construed as a potential conflict of interest.
